# The GBS PI-2a Pilus Is Required for Virulence in Mice Neonates

**DOI:** 10.1371/journal.pone.0018747

**Published:** 2011-04-15

**Authors:** Salvatore Papasergi, Sara Brega, Michel-Yves Mistou, Arnaud Firon, Virginie Oxaran, Ron Dover, Giuseppe Teti, Yechiel Shai, Patrick Trieu-Cuot, Shaynoor Dramsi

**Affiliations:** 1 Institut Pasteur, Unité de Biologie des Bactéries Pathogènes à Gram-Positif, Paris, France; 2 URA CNRS 2172, Paris, France; 3 The Elie Metchnikoff Deparment, University of Messina, Messina, Italy; 4 INRA, MICALIS UMR 1319, Equipe Paroi, Jouy-en-Josas, France; 5 INRA, MICALIS UMR1319, Equipe Protéines de Surface Utiles, Jouy-en-Josas, France; 6 Depatment of Biological Chemistry, The Weizmann Institute of Science, Rehovot, Israel; University of California Merced, United States of America

## Abstract

**Background:**

*Streptococcus agalactiae* (Group B Streptococcus) is a leading cause of sepsis and meningitis in newborns. Most bacterial pathogens, including gram-positive bacteria, have long filamentous structures known as pili extending from their surface. Although pili are described as adhesive organelles, they have been also implicated in many other functions including thwarting the host immune responses. We previously characterized the pilus-encoding operon PI-2a (*gbs1479-1474*) in strain NEM316. This pilus is composed of three structural subunit proteins: PilA (Gbs1478), PilB (Gbs1477), and PilC (Gbs1474), and its assembly involves two class C sortases (SrtC3 and SrtC4). PilB, the *bona fide* pilin, is the major component whereas PilA, the pilus associated adhesin, and PilC the pilus anchor are both accessory proteins incorporated into the pilus backbone.

**Methodology/Principal Findings:**

In this study, the role of the major pilin subunit PilB was tested in systemic virulence using 6-weeks old and newborn mice. Notably, the non-piliated *ΔpilB* mutant was less virulent than its wild-type counterpart in the newborn mice model. Next, we investigated the possible role(s) of PilB in resistance to innate immune host defenses, i.e. resistance to macrophage killing and to antimicrobial peptides. Phagocytosis and survival of wild-type NEM316 and its isogenic *ΔpilB* mutant in immortalized RAW 264.7 murine macrophages were not significantly different whereas the isogenic Δ*sodA* mutant was more susceptible to killing. These results were confirmed using primary peritoneal macrophages. We also tested the activities of five cationic antimicrobial peptides (AMP-1D, LL-37, colistin, polymyxin B, and mCRAMP) and found no significant difference between WT and *ΔpilB* strains whereas the isogenic *dltA* mutant showed increased sensitivity.

**Conclusions/Significance:**

These results question the previously described role of PilB pilus in resistance to the host immune defenses. Interestingly, PilB was found to be important for virulence in the neonatal context.

## Introduction

*Streptococcus agalactiae* (also referred to as Group B Streptococcus, GBS) is a gram-positive encapsulated bacterium responsible for life-threatening infections in newborns, elderly, and adults with underlying diseases [1], [2]. Two distinct clinical syndromes, early-onset disease (EOD) or late-onset disease (LOD) have been described in neonates and young infants [3]. For EOD, the main route of infection is assumed to be a vertical transmission from inhalation during parturition *of S. agalactiae*-contaminated vaginal or amniotic fluid, resulting in subsequent systemic infection after translocation across the respiratory epithelium. For LOD, the mode of transmission and the infection route still remains unclear. Once into the bloodstream, *S. agalactiae* can cause septicemia and then cross the blood-brain barrier to cause meningitis.

Bacterial pili have recently been recognized in major human pathogens such as *S. agalactiae*, *Streptococcus pyogenes* (GAS), and *Streptococcus pneumoniae* (for reviews see [4], [5], [6], [7], [8], [9]). Sortase-mediated pilus assembly was first demonstrated in *Corynebacterium diphtheriae* [10], [11] and these pioneer studies revealed the existence of three conserved motifs within the major pilin subunit that are necessary for pilus formation: i) the pilin motif (WxxxVxVYPK); ii) the E-box domain (YxLxETxAPxGY); and iii) the cell wall sorting signal (LPxTG followed by a hydrophobic domain and a positively charged tail). The current model for pilus assembly is as follows: the major subunit is assembled to form a pilus by a *cis*-encoded sortase that catalyzes the covalent attachment of the conserved lysyl residue of the pilin motif of one subunit with the conserved threonyl residue of the LPxTG motif of another subunit. In addition, one or more accessory subunits are incorporated into the pilus by an as yet unknown mechanism that requires the pilus-specific sortase. The crystallographic structures of two major pilins have now shown that the E-box domain is involved in the formation of intramolecular isopeptide bond conferring higher stability to the pilin monomer [12], [13]. Then, during the final step, the pilus fiber is covalently linked to the peptidoglycan by either the pilus-specific or the housekeeping sortase. This mechanism of pilus assembly catalyzed by class C sortases has now been characterized in several gram-positive pathogens using similar genetic and biochemical analyses [14], [15], [16], [17], [18], [19], [20], [21], [22].

Three genomic loci (PI-1, PI-2a, and PI-2b) have been described in GBS strains [23], the latter two being mutually exclusive as they are located at the same chromosomal location. In a survey of 289 GBS clinical isolates, PI-1, PI-2a, and PI-2b were detected in 72%, 73%, and 27% of the strains, the combination of PI-1 + PI-2a being the most frequent [24]. We and others previously carried out a detailed structural and functional analysis of the pilus locus PI-2a (*gbs1479–1474*) in GBS strain NEM316 [16], [23]. This locus encodes the three structural pilus subunits PilA (Gbs1478), PilB (Gbs1477), and PilC (Gbs1474) whose assembly involves two class C sortases (SrtC3 and SrtC4). PilB, the *bona fide* pilin, is the major component; PilC is a minor associated component mainly localized at the base of the pilus [25]; and PilA is the adhesin located at intervals along the pilus backbone [16]. The PI-2a GBS pili have also been implicated in mediating attachment to human epithelial cells [16], [26], [27], in biofilm formation [26], [28], in the adhesion and invasion of brain microvascular endothelial cells [29], and in promoting transepithelial migration [30].

Intriguingly, the pilin subunit PilB of PI-2a was also reported to mediate resistance to cathelicidin antimicrobial peptide and phagocyte killing, to increase bloodstream survival, and to confer virulence in a mouse challenge model [31].

Here, we re-investigate the contribution of PilB in the virulence of strain NEM316 using two different mice models and in resistance to innate host immune defenses by testing GBS survival to killing by macrophages or antimicrobial peptides.

## Results

### PilB mutant is attenuated for virulence in a neonatal mouse infection model

**Figure 1:**
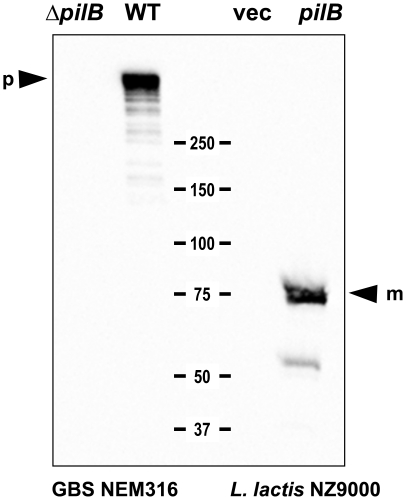
Surface expression of pilB in *S. agalactiae* and in recombinant *L. lactis* strains. Immunoblots of cell-wall protein extracts of GBS and recombinant *L. lactis* strains with the antiserum against PilB. Numbers indicate the size of molecular weight marker in kDa. The positions of the monomeric (m) and polymeric (p) form of PilB are marked by arrowheads.

To investigate the role of the pilus in invasive disease, we made use of the previously described in-frame deletion mutant of *gbs1477*, encoding the backbone protein PilB in GBS strain NEM316 [16] in combination with the heterologous expression of PilB under the constitutive lactococcal p23 promoter in the non-pathogenic host *Lactococcus lactis* strain NZ9000. As shown by Western blotting using anti-PilB polyclonal antibody, expression of *pilB* in *L. lactis* strain NZ9000 was associated with the presence in the cell wall extracts of a band of 75 kDa corresponding to PilB monomer that was missing in the control strain *L. lactis* harboring the cloning vector without DNA insert (Fig. 1). As previously shown [16], PilB appears mainly as a polymer in GBS strain NEM316 (Fig. 1) whereas PilB monomers are directly anchored to the cell wall in *L. lactis*.

**Figure 2:**
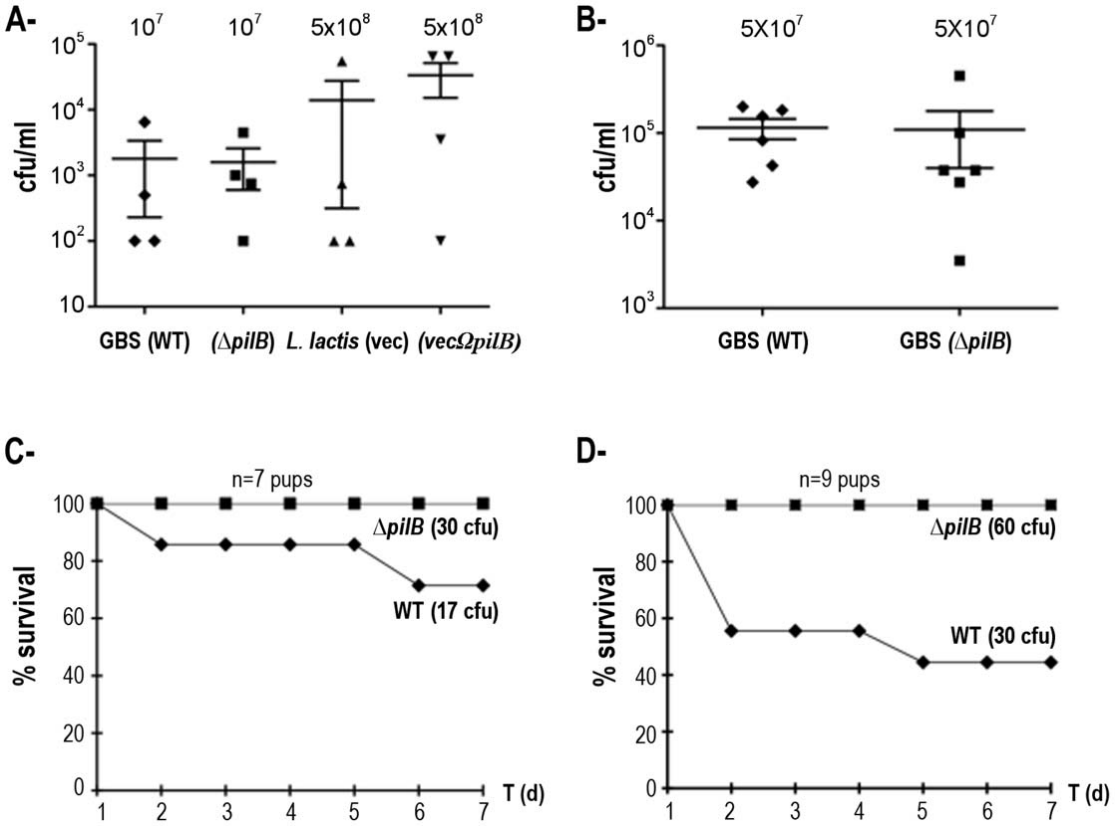
Role of PilB in virulence. A- Bacterial loads in blood of 6-weeks CD1 mice collected 24 h post-intravenous injection with GBS or recombinant *L. lactis* strains (4 mice per strain). B- Similar experiment than in A with higher GBS inoculum (6 mice per strain). C- Survival index of newborn mice infected intraperitoneally with lower inoculum (7 pups per strain). D- Same experiment as in C with higher inoculum (9 pups per strain).

*S. agalactiae* and lactococcal strains were then tested in parallel for 6-weeks old CD1 mice were challenged intravenously with two doses (10^7^ or 5×10^7^ CFU) of GBS NEM316 and *ΔpilB* mutant, and with a higher dose (5×10^8^ CFU) of *L. lactis* strains NZ9000 expressing or not *pilB*, and blood was collected 24 h later to enumerate viable bacterial CFUs. As shown in Fig. 2A and 2B, no significant differences were found between strains expressing or not *pilB* in these conditions. It is worth noting that the inter-animal variability is quite important in these experiments.

We then tested the role of PilB in a neonatal sepsis model. BALB/c mice (≤24 h-old) were infected subcutaneously with low (17 to 30 CFU) and high (30 to 60 CFU) GBS challenge inoculum (Fig. 2C and 2D, respectively). At low dose, only 60% of the mice infected with 17 CFU of WT strain survived whereas 100% of mice infected with 30 CFU of *ΔpilB* mutant survived (Fig 2C). More strikingly, only 40% of the mice infected with 30 CFU of WT strain survived whereas all the mice survived when infected with 60 CFU of *ΔpilB* mutant (Fig. 2D). Thus in both experiments, we consistently observed that a higher number of mice survived when challenged with *ΔpilB* mutant as compared to WT strain after 24 h post-infection demonstrating the importance of PilB pilus of *S. agalactiae* NEM316 in the neonatal context.

### PilB does not promote bacterial survival in murine macrophages

**Figure 3:**
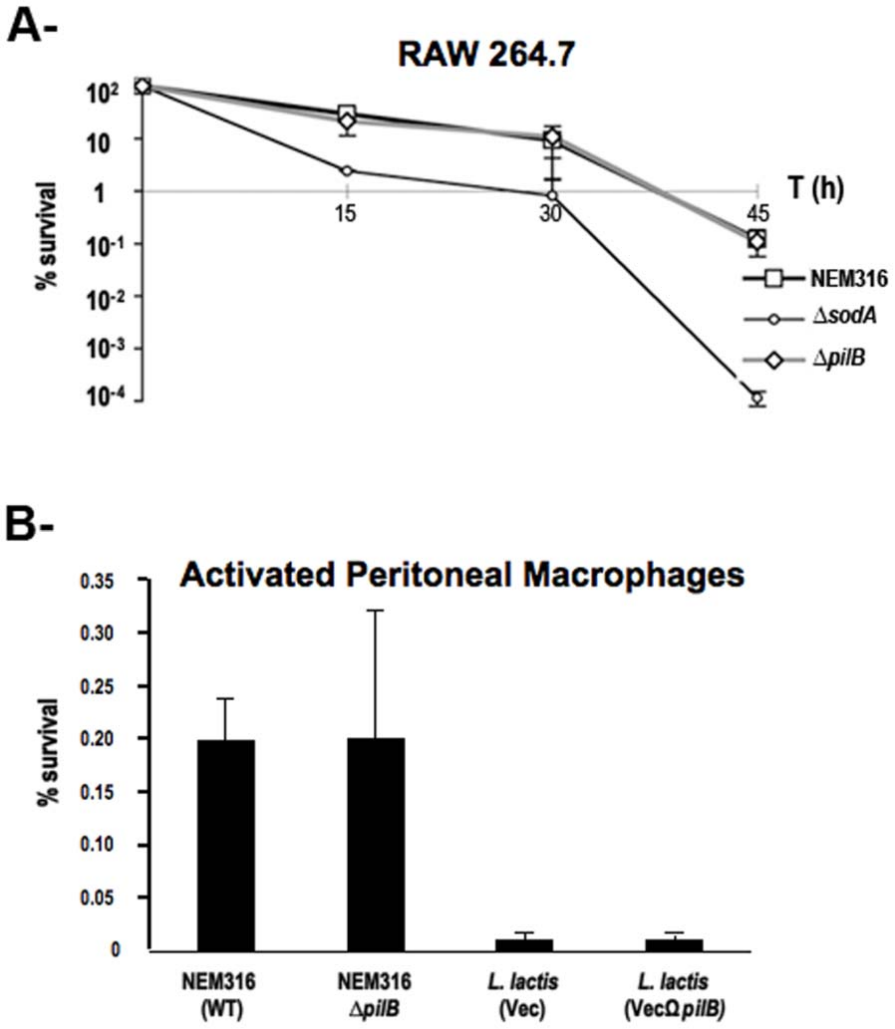
Bacterial survival in cultured and primary murine macrophages. A- Immortalized RAW264.7 were cultured *in vitro* and exposed to *S. agalactiae* NEM316 (WT), the non-piliated mutant (*ΔpilB*), and the *sodA* mutant (NEM1640) at an m.o.i. of 10 bacteria per cell in 24-wells plate. Error bars represent the standard deviation of three independent experiments done in duplicate for each strain studied. B- Survival of *S. agalactiae* and *L. lactis* strains in thioglycolate-elicitated primary murine peritoneal macrophages. Bacteria in exponential phase (OD_600_ of about 0.6) were added to macrophages (m.o.i. of 10) for 30 min and then survival was measured after 2 h30 at 37°C. Error bars represent the standard deviation of a representative experiment done in triplicate for each strain studied.

The survival of GBS strain NEM316 and its isogenic non-piliated mutant *ΔpilB* was compared in the widely used murine macrophage cell line RAW 264.7. We also included as control the NEM316*ΔsodA* mutant which was previously shown to display increased susceptibility to bacterial killing by macrophages [32]. As shown in Fig. 3A, the wild-type strain NEM316 (WT) and the *ΔpilB* mutant displayed a similar survival kinetic in this phagocytic cell line. In contrast, survival of the isogenic *ΔsodA* mutant was significantly reduced compared to the wild-type strain.

Since phagocytic cell lines are considered to be less harmful for bacteria than primary macrophages, we performed similar experiments using thioglycolate-elicitated murine peritoneal macrophages. *S. agalactiae* and lactococcal strains were tested in parallel for survival in murine peritoneal macrophages (Fig. 3B). Whereas piliated *S. agalactiae* NEM316 WT and non-piliated *ΔpilB* mutant survived similarly, a dramatic phagocytic killing of both lactococcal strains was observed (20-fold decreased as compared to GBS strains). No gain of function was noticed for the *L. lactis* strain expressing *pilB* when compared to the *L. lactis* strain expressing the vector alone.

Altogether these results indicate that NEM316 PilB is not involved in resistance to bacterial killing by macrophages.

### PilB does not confer resistance to antimicrobial peptides

**Figure 4:**
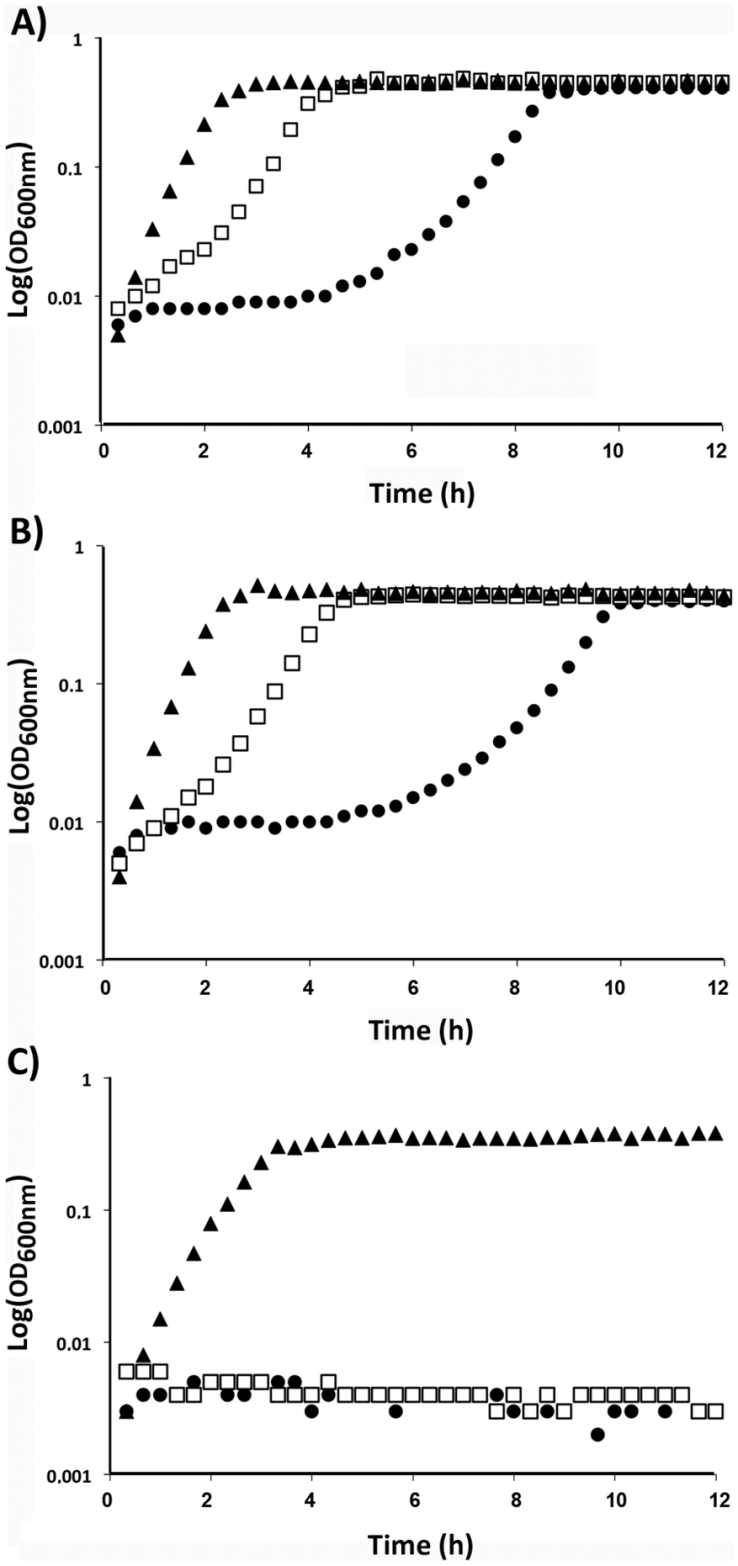
Effect of the murine cathelicidin mCRAMP on bacterial growth. Prewarmed TH broth buffered with HEPES (100 mM) and containing or not mCRAMP was inoculated with overnight NEM316 (A), NEM316Δ*pilB* (B), and NEM316*ΔdltA* (C) to give approximately 10^7^ cfu/ml. The inoculated broth was distributed (150 µl) in 96 wells plate incubated at 37°C with constant shaking in a plate reader and the OD_600__nm_ was recorded every 20 minutes for 12 hours. Blank values (TH-HEPES) were subtracted from experimental values to eliminate background readings. ▴ TH-HEPES medium without peptide (sterile water was added instead); □ and 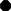 TH-Hepes medium containing 100 and 200 µg/ml of mCRAMP respectively. These results are representative of three independent experiments.

It has been shown by others that GBS NCTC10/84*ΔpilB* exhibited enhanced susceptibility to various cationic antimicrobial peptides CAMPs (mCRAMP, polymyxin B, and LL-37). We thus tested the activities of four cationic molecules having similar net charges such as AMP-1D (+6), LL-37 (+6), colistin (+5) and polymyxin B (+5) by determining the minimal inhibitory concentration required to inhibit the growth of 90% of the bacteria (MIC_90_). As shown in Table 1, no difference was found between GBS WT and the isogenic *ΔpilB* mutant. In contrast, the *dltA* mutant showed increased sensitivity to these CAMPs, as shown previously [33]. For consistency with our animal models, we also tested the effect of various concentrations of the murine cathelicidin mCRAMP (+6) on the growth curve of WT, *ΔpilB*, and *ΔdltA* in TH medium (Fig. 4). Again, no significant difference was found between WT and the *ΔpilB* mutant. Similar results were obtained using this experimental condition for the three other tested CAMPs: colistine, AMP-1D, and polymyxin B (data not shown). We also observed that expression of *pilB* in *L. lactis* NZ9000 did not modify the MIC_90_ towards these antibiotics (Table 1), nor the bacterial growth (Fig. S1). Collectively, our results do not support a role for the pilus in resistance to cationic antimicrobial peptides.

**Table 1:** Minimal Inhibitory Concentrations (MIC_90_) of various antimicrobial peptides towards GBS strain NEM316 (WT) and its derivatives and *L. lactis* NZ9000 expressing or not the pilus backbone subunit encoding gene *pilB* from the multicopy vector pOri23.

	*S. agalactiae*				*L. lactis*	
	WT	*ΔpilB*	*ΔdltA*		vec	vecΩ*pilB*
AMP-1D	8	8	4		4	4
LL-37	>64	>64	16		16	16
Colistin	>256	>256	16		>256	>256
Polymyxin B	128	128	8		>256	>256

Values are expressed in µg/ml.

## Discussion

The goal of the present work was to evaluate the contribution of the pilin subunit PilB of the GBS pilus-encoding operon PI-2a to bacterial virulence. This was done either by deleting the corresponding gene *pilB* in the WT serotype III strain NEM316 or by expressing it in the food grade bacterium *L. lactis* NZ9000. In a 6 weeks old CD1 septicemic mouse model, we observed that PilB was dispensable for bacterial virulence in both genetic backgrounds. These results conflict with those of Maisey *et al*. (2008) who reported that PilB of GBS NCTC10/84, a highly hemolytic serotype V strain [34], conferred virulence to the parental GBS strain and to *L. lactis*, as assessed in a similar animal model. Moreover, over-expression of the *pilB* gene alone in the non-pathogenic *L. lactis* was found to enhance resistance to phagocyte killing, increased bloodstream survival, and conferred virulence in a mouse model [31]. The latter observation was intriguing as it suggested that PilB is an essential GBS virulence factor, being sufficient to turn the unencapsulated and non-pathogenic bacterium *L. lactis* into an invasive extracellular bacteria.

The molecular basis of PilB-associated virulence is thought to reside in its ability to confer resistance to CAMP and phagocytosis, and in consequence to bloodstream survival. To exert antimicrobial activity, CAMPs must bind to the bacterial surface, whether they act by the inhibition of biosynthetic processes on the bacterial surface, pore formation in the cytoplasmic membrane, or yet other mechanisms. The bacterial surface is negatively charged owing to the production of anionic polymers. Moreover, the outer and inner leaflets of the bacterial cytoplasmic membrane are also negatively charged. In Gram-positive bacteria, resistance to CAMPs is mainly due to an increase of the positive surface charge through increase in D-alanylation of the LTAs or incorporation of L-lysine into membrane phosphatidylglycerol, more rarely to specific proteolytic degradation [35]. PilB from GBS strains NEM316 and NCTC10/84 display 84.5% of sequence identity but the pilin subunit from NEM316 is positively charged (+2; pKi 8.44) at neutral pH whereas that from NCTC10/84 is slightly electronegative (−2; pKi 6.57). These observations, combined with the fact that GBS pili are not evenly distributed along the bacterial surface, strongly argue against their involvement in CAMP resistance by electrostatic repulsion and it is worth noting that neither proteins contain any known proteolytic domain. Consistently, we observed that NEM316 PilB did not modulate the susceptibility of the piliated original host nor that of *L. lactis* towards 5 CAMPs including mCRAMP, whereas the NEM316 Δ*dltA* mutant displayed increased susceptibility towards all tested molecules.

We also demonstrated that NEM316 PilB is dispensable for entry and survival within two different mouse-derived macrophage-like cell lines, RAW 264.7 (Fig. 3A) and MH-S of alveolar origin (data not shown), in non-opsonic conditions. Macrophages respond to infection or injury by changing from a « resting » cellular phenotype to an « activated » state defined by the expression of various cytotoxic effector molecules. Regulation of the transition from a resting to an activated state is effected by cytokines and/or pathogenic signals. Thioglycolate is often used to induce peritonitis in mice to recover primary peritoneal macrophages. We thus tested survival of wild-type GBS strain NEM316 and its isogenic *ΔpilB* mutant in this cellular model mimicking *in vivo* situation and, again, did not found any significant differences between WT and mutant strains (Fig. 3). Heterologous expression of *pilB* from NEM316 in *L. lactis* did not confer any significant advantage for survival in primary activated macrophages, as compared to the control strain (Fig. 3). In conclusion, our results do not substantiate the proposal that PilB is a major player in resistance to innate immune host defenses, i.e. resistance to macrophage killing and to antimicrobial peptides. However, we cannot exclude that phenotypic differences are not due to sequence variations in the PilB proteins.

As natural GBS infections primarily occur in the neonatal context, we compared the virulence of NEM316 WT and *ΔpilB* strains in newborn BALB/c mice and found that the *ΔpilB* was significantly less virulent than the wild-type NEM316 strain (Fig. 1CD). Thus, NEM316 PilB appears to contribute to virulence in neonate but not in adult mice. In this work, it is worth mentioning that newborn and adult mice were infected subcutaneously and intravenously, respectively. However, we consider unlikely that the observed difference in virulence reflects the use of different modes of injection as, in our experience, adult mice are highly sensitive to a GBS challenge administered i.v. Indeed, we have infected subcutaneously two- and three- months old adult mice (groups of 12 mice) with high doses of GBS NEM316, ranging from 2.5×10^8^ to 10^9^ cfu, and no mortality was observed (data not shown).

Besides having an immature immune system, newborn are also more permissive to infections than adult mice due to increased bacterial translocation through the epithelia of their major organs, such as intestine, lung, kidney, liver, and brain [36], [37]. The GBS PI-2a pilus operon was reported to promote biofilm formation, adherence to human epithelial cells and transepithelial migration, and adhesion and invasion of brain microvascular endothelial cells. These phenotypic traits may therefore strongly impact GBS virulence in neonate mice but not in adult mice, as observed in this study. Our results also illustrate the fact that the definition of a virulence factor primarily depends on the animal model used. Hence, GBS pilus can be considered as a virulence factor in the neonatal context.

## Materials and Methods

### Ethics statement

All of the animal experiments described in the present study were conducted at the Metchnikoff Department of the University of Messina according to the European Union guidelines for the handling of laboratory animals (http://ec.europa.eu/environment/chemicals/lab_animals/home_en.html) and were approved by the relevant national authority (Istituto Superiore di Sanità of Italy).

### Bacterial strains, media, and growth conditions

*Streptococcus agalactiae* NEM316 was responsible for a fatal septicaemia and belongs to the capsular serotype III. The complete genome sequence of this strain has been determined [38]. *S. agalactiae* were grown in Todd-Hewitt (TH) medium (Difco-BD). *Lactococcus lactis* strain NZ9000 [39] was grown in M17 medium supplemented with 1% glucose (GM17). For antibiotic selection in *L. lactis*, 5 µg/ml erythromycin was added to GM17. Heterologous expression of *pilB* in *L. lactis* strain was realized as follows: The full-lengh *pilB* gene was amplified from genomic DNA of strain NEM316 and subcloned in the lactococcal vector pIL253::P23 [40], a high copy number erythromycin resistance plasmid expressing the cloned gene from the strong constitutive promoter P23 [41]. The primers used for *pilB* amplification were: 1477a (5′-ATG GGC CCA TGA AAA AAA TCA ACA AAT GTC TTA CAG T – 3′) and 1477b (5′ - ATA CTG CAG CCT AAA TAA TGG CTC TTG CTT ATG – 3′). The 2.1-kb PCR product was then cut by *ApaI* and *PstI* (New England Biolabs), purified, and cloned into pIL253::P23 cut with the same enzymes resulting in pIL253::P23Ω*gbs1477* (pVE5616). This ligation mixture was transformed to electrocompetent *L. lactis* IL1403. After verification by DNA sequencing, pVE5616 was transferred into *L. lactis* NZ9000.

### General DNA techniques

Standard recombinant techniques were used for nucleic acid cloning and restriction analysis [42]. Plasmid DNA from *E. coli* was prepared by rapid alkaline lysis using the Nucleospin Plasmid kit (Macherey-Nagel). Genomic DNA from *S. agalactiae* was prepared using the DNeasy Blood and Tissue kit (Qiagen). PCR was carried out with the High-Fidelity Phusion DNA polymerase as recommended by the manufacturer (Finnzymes).

### Immunoblotting

Cell wall protein extracts were prepared by harvesting 50 ml of bacteria in early stationary phase (0D_600_ = 1). The bacterial pellet was washed once in Tris-HCl buffer (50 mM, pH 7.3) and once in the mutanolysin digestion buffer (Tris-HCl 50 mM pH 7.3 supplemented with 20% sucrose and a complete protease inhibitor cocktail (Roche)). Mutanolysin (Sigma) dissolved to 5,000 U ml^−1^ in potassium phosphate buffer (pH 6.2) was then added to the bacterial suspension to give a final concentration of 100 U ml^−1^ and samples were rotated for 2 h at 37°C. After centrifuging at 13,000 g for 15 min at 4°C, supernatants corresponding to the cell wall extracts were analyzed on SDS-PAGE or kept frozen at −20°C. For western blotting, proteins were boiled in Laemmli sample buffer, resolved on Tris-Acetate Criterion XT gradient gels 4–12% SDS-PAGE gels (BioRad) and transferred to nitrocellulose membrane (Hybond-C, Amersham). PilB was detected using polyclonal antibodies and horseradish peroxydase (HRP)-coupled anti-rabbit secondary antibodies (Zymed) and the Western pico chemiluminescence kit (Thermo scientific).

### Adult mouse infection model

Two groups of male CD-1 mice (6- weeks old) were injected intravenously (i.v.) *via* the tail vein with the indicated amounts of early logarithmic-phase of GBS or *L. lactis* strains. Mice were monitored daily for survival. Bacteremia was assessed at 24 h by blood collection and enumeration of CFUs on TH or M17 agar plates.

### Neonatal mouse model of GBS sepsis

Neonatal *BALB/c* mice were used for virulence studies. Randomized groups of 7 to 10 mice pups were inoculated subcutaneously with a dilution of mild-log-phase strain NEM316 or *ΔpilB* (0.03 ml of each strain in 0.9% NaCl). Under these condition (from 17 to 60 CFU/mouse), GBS are contained at the inoculation site or spread systematically, depending on the bacterial intrinsic virulence factor and the ability of the host immune system to prevent bacterial grow. Mice were observed daily for up 8 days post-infection. In this model, deaths are rarely observed after Day 5.

### Assay for GBS intracellular survival in macrophages

Cells were infected at a multiplicity of infection (m.o.i.) of 10 bacteria per cell for 1 h at 37°C in 10% of CO_2._ The monolayers were then washed four times with PBS and fresh medium containing gentamicin (100 µg/ml) was added to kill extracellular bacteria (time zero of the assay). To quantify intracellular GBS at different times of post-infection, the supernatants were removed and the cells were disrupted by the addition of 1 ml sterile deionized ice-cold water and repeated pipetting. Serial dilutions of the lysate were plated on TH agar for counting of viable bacteria. Survival index was calculated as follows: (CFU on plate count/CFU in original inoculum)×100. Assays were performed in duplicate and were repeated at least three times.

### Determination of the Minimal Inhibitory Concentration (MIC)

The MICs (µg/ml) of *S. agalactiae* towards antimicrobial peptides (AMP-1D, LL-37, colistin, and polymyxin B) were performed by a dilution method in 96 wells polypropylene microplates (Costar, Cambridge, MA) containing Todd-Hewitt (TH) broth buffered with 100 mM HEPES using two biological replicates in duplicate. Polymyxin B and colistin were purchased from Sigma whereas LL-37 and AMP-1D were synthesized in the Department of Biological Chemistry (Rehovot, Israel). Bacteria (approx. 10^5^ CFU) were added to wells containing increasing concentrations of the antimicrobial peptides. Plates were incubated overnight at 37°C and were then read at OD_600 nm_ with a microplate reader BioTek Synergy for bacterial growth. The MIC_90_ was considered and expressed as the lowest peptide concentration inhibiting growth of 90% of the bacterial cells. The same experimental procedure was used for *L. lactis* except that bacteria were grown at 30°C in M17 broth supplemented with glucose (1%) and erythromycin (5 µg/ml).

### Growth curves in the presence of mCRAMP

Overnight cultures of GBS in TH broth buffered with HEPES (100 mM) were diluted in fresh media to give approximately 10^7^ cfu/ml and 150 µl were distributed in 96 wells plate without (control wells) or with mCRAMP at selected concentrations (test wells). The microplate was incubated at 37°C with constant shaking in the BioTek Synergy plate reader and the OD_600__nm_ was recorded every 20 min for 12 h. The same experimental procedure was used for *L. lactis* except that bacteria were grown at 30°C in M17 broth supplemented with glucose (1%) and erythromycin (5 µg/ml). mCRAMP was purchased from TEBU.

## Supporting Information

Figure S1 **Effect of the murine cathelicidin mCRAMP on bacterial growth.** Overnight culture of the control strain *L. lactis* NZ9000/vec (A) or the *L. lactis* NZ9000/vecΩ*pilB* strain expressing the GBS PilB protein (B) were diluted in M17 broth supplemented with glucose (1%) and erythromycin (5 µg/ml) to give approximately 10^6^ cfu/ml. The inoculated broths were distributed (150 µl) in 96 wells plate incubated at 30°C with constant shaking in a plate reader and the OD_600__nm_ was recorded every 20 minutes for 12 hours. Blank values (M17-glucose-erythromycin) were subtracted from experimental values to eliminate background readings. ▴, M17-glucose-erythromycin medium without peptide (sterile water was added instead); ▪, presence of the mCRAMP drug at 5 (light blue), 10 (violet), 20 (green), and 40 (red) µg/ml, respectively. These results are representative of three independent experiments.
(DOCX)

## References

[1] Phares CR, Lynfield R, Farley MM, Mohle-Boetani J, Harrison LH (2008). Epidemiology of invasive group B streptococcal disease in the United States, 1999-2005.. JAMA.

[2] Poyart C, Reglier-Poupet H, Tazi A, Billoet A, Dmytruk N (2008). Invasive group B streptococcal infections in infants, France.. Emerg Infect Dis.

[3] Costakos DT, Walden J (2009). CDC changes recommendation for babies born to mothers with Streptococcus agalactiae.. WMJ.

[4] Mandlik A, Swierczynski A, Das A, Ton-That H (2008). Pili in Gram-positive bacteria: assembly, involvement in colonization and biofilm development.. Trends Microbiol.

[5] Proft T, Baker EN (2009). Pili in Gram-negative and Gram-positive bacteria - structure, assembly and their role in disease.. Cell Mol Life Sci.

[6] Scott JR, Zahner D (2006). Pili with strong attachments: Gram-positive bacteria do it differently.. Mol Microbiol.

[7] Telford JL, Barocchi MA, Margarit I, Rappuoli R, Grandi G (2006). Pili in gram-positive pathogens.. Nat Rev Microbiol.

[8] Ton-That H, Schneewind O (2004). Assembly of pili in Gram-positive bacteria.. Trends Microbiol.

[9] Kreikemeyer B, Gamez G, Margarit I, Giard JC, Hammerschmidt S (2010). Genomic organization, structure, regulation and pathogenic role of pilus constituents in major pathogenic Streptococci and Enterococci..

[10] Ton-That H, Marraffini LA, Schneewind O (2004). Sortases and pilin elements involved in pilus assembly of Corynebacterium diphtheriae.. Mol Microbiol.

[11] Ton-That H, Schneewind O (2003). Assembly of pili on the surface of Corynebacterium diphtheriae.. Mol Microbiol.

[12] Budzik JM, Poor CB, Faull KF, Whitelegge JP, He C (2009). Intramolecular amide bonds stabilize pili on the surface of bacilli.. Proc Natl Acad Sci U S A.

[13] Kang HJ, Coulibaly F, Clow F, Proft T, Baker EN (2007). Stabilizing isopeptide bonds revealed in gram-positive bacterial pilus structure.. Science.

[14] Barocchi MA, Ries J, Zogaj X, Hemsley C, Albiger B (2006). A pneumococcal pilus influences virulence and host inflammatory responses.. Proc Natl Acad Sci U S A.

[15] Budzik JM, Marraffini LA, Schneewind O (2007). Assembly of pili on the surface of Bacillus cereus vegetative cells.. Mol Microbiol.

[16] Dramsi S, Caliot E, Bonne I, Guadagnini S, Prevost MC (2006). Assembly and role of pili in group B streptococci.. Mol Microbiol.

[17] Hendrickx AP, Bonten MJ, van Luit-Asbroek M, Schapendonk CM, Kragten AH (2008). Expression of two distinct types of pili by a hospital-acquired Enterococcus faecium isolate.. Microbiology.

[18] LeMieux J, Hava DL, Basset A, Camilli A (2006). RrgA and RrgB are components of a multisubunit pilus encoded by the Streptococcus pneumoniae rlrA pathogenicity islet.. Infect Immun.

[19] Mandlik A, Swierczynski A, Das A, Ton-That H (2007). Corynebacterium diphtheriae employs specific minor pilins to target human pharyngeal epithelial cells.. Mol Microbiol.

[20] Mora M, Bensi G, Capo S, Falugi F, Zingaretti C (2005). Group A Streptococcus produce pilus-like structures containing protective antigens and Lancefield T antigens.. Proc Natl Acad Sci U S A.

[21] Nallapareddy SR, Singh KV, Sillanpaa J, Garsin DA, Hook M (2006). Endocarditis and biofilm-associated pili of Enterococcus faecalis.. J Clin Invest.

[22] Sillanpaa J, Prakash VP, Nallapareddy SR, Murray BE (2009). Distribution of genes encoding MSCRAMMs and Pili in clinical and natural populations of Enterococcus faecium.. J Clin Microbiol.

[23] Rosini R, Rinaudo CD, Soriani M, Lauer P, Mora M (2006). Identification of novel genomic islands coding for antigenic pilus-like structures in Streptococcus agalactiae.. Mol Microbiol.

[24] Margarit I, Rinaudo CD, Galeotti CL, Maione D, Ghezzo C (2009). Preventing bacterial infections with pilus-based vaccines: the group B streptococcus paradigm.. J Infect Dis.

[25] Nobbs AH, Rosini R, Rinaudo CD, Maione D, Grandi G (2008). Sortase A utilizes an ancillary protein anchor for efficient cell wall anchoring of pili in Streptococcus agalactiae.. Infect Immun.

[26] Konto-Ghiorghi Y, Mairey E, Mallet A, Dumenil G, Caliot E (2009). Dual role for pilus in adherence to epithelial cells and biofilm formation in Streptococcus agalactiae.. PLoS Pathog.

[27] Krishnan V, Gaspar AH, Ye N, Mandlik A, Ton-That H (2007). An IgG-like domain in the minor pilin GBS52 of Streptococcus agalactiae mediates lung epithelial cell adhesion.. Structure.

[28] Rinaudo CD, Rosini R, Galeotti CL, Berti F, Necchi F (2010). Specific involvement of pilus type 2a in biofilm formation in group B Streptococcus.. PLoS One.

[29] Maisey HC, Hensler M, Nizet V, Doran KS (2007). Group B streptococcal pilus proteins contribute to adherence to and invasion of brain microvascular endothelial cells.. J Bacteriol.

[30] Pezzicoli A, Santi I, Lauer P, Rosini R, Rinaudo D (2008). Pilus backbone contributes to group B Streptococcus paracellular translocation through epithelial cells.. J Infect Dis.

[31] Maisey HC, Quach D, Hensler ME, Liu GY, Gallo RL (2008). A group B streptococcal pilus protein promotes phagocyte resistance and systemic virulence.. FASEB J.

[32] Poyart C, Pellegrini E, Gaillot O, Boumaila C, Baptista M (2001). Contribution of Mn-cofactored superoxide dismutase (SodA) to the virulence of Streptococcus agalactiae.. Infect Immun.

[33] Poyart C, Pellegrini E, Marceau M, Baptista M, Jaubert F (2003). Attenuated virulence of Streptococcus agalactiae deficient in D-alanyl-lipoteichoic acid is due to an increased susceptibility to defensins and phagocytic cells.. Mol Microbiol.

[34] Doran KS, Liu GY, Nizet V (2003). Group B streptococcal beta-hemolysin/cytolysin activates neutrophil signaling pathways in brain endothelium and contributes to development of meningitis.. J Clin Invest.

[35] Peschel A (2002). How do bacteria resist human antimicrobial peptides?. Trends Microbiol.

[36] Burns-Guydish SM, Olomu IN, Zhao H, Wong RJ, Stevenson DK (2005). Monitoring age-related susceptibility of young mice to oral Salmonella enterica serovar Typhimurium infection using an in vivo murine model.. Pediatr Res.

[37] Urao M, Teitelbaum DH, Drongowski RA, Coran AG (1996). The association of gut-associated lymphoid tissue and bacterial translocation in the newborn rabbit.. J Pediatr Surg.

[38] Glaser P, Rusniok C, Buchrieser C, Chevalier F, Frangeul L (2002). Genome sequence of Streptococcus agalactiae, a pathogen causing invasive neonatal disease.. Mol Microbiol.

[39] Eichenbaum Z, Federle MJ, Marra D, de Vos WM, Kuipers OP (1998). Use of the lactococcal nisA promoter to regulate gene expression in gram-positive bacteria: comparison of induction level and promoter strength.. Appl Environ Microbiol.

[40] Piard JC, Hautefort I, Fischetti VA, Ehrlich SD, Fons M (1997). Cell wall anchoring of the Streptococcus pyogenes M6 protein in various lactic acid bacteria.. J Bacteriol.

[41] van der Vossen JM, van der Lelie D, Venema G (1987). Isolation and characterization of Streptococcus cremoris Wg2-specific promoters.. Appl Environ Microbiol.

[42] Sambrook J, Fritsch EF, Maniatis T (1989). Molecular cloning: a laboratory manual, Second Edition..

