# Supplementary material for: The GBS PI-2a Pilus Is Required for Virulence in Mice Neonates
Source: PLoS One. 2011 Apr 15;6(4):e18747. doi: 10.1371/journal.pone.0018747 (PMC3078112; doi:10.1371/journal.pone.0018747)
Supplement: Figure S1 — Effect of the murine cathelicidin mCRAMP on bacterial growth. Overnight culture of the control strain L. lactis NZ9000/vec (A) or the L. lactis NZ9000/vecΩpilB strain expressing the GBS PilB protein (B) were diluted in M17 broth supplemented with glucose (1%) and erythromycin (5 µg/ml) to give approximately 106 cfu/ml. The inoculated broths were distributed (150 µl) in 96 wells plate incubated at 30°C with constant shaking in a plate reader and the OD600 nm was recorded every 20 minutes for 12 hours. Blank values (M17-glucose-erythromycin) were subtracted from experimental values to eliminate background readings. ▴, M17-glucose-erythromycin medium without peptide (sterile water was added instead); ▪, presence of the mCRAMP drug at 5 (light blue), 10 (violet), 20 (green), and 40 (red) µg/ml, respectively. These results are representative of three independent experiments. (DOCX) [file pone.0018747.s001.docx]

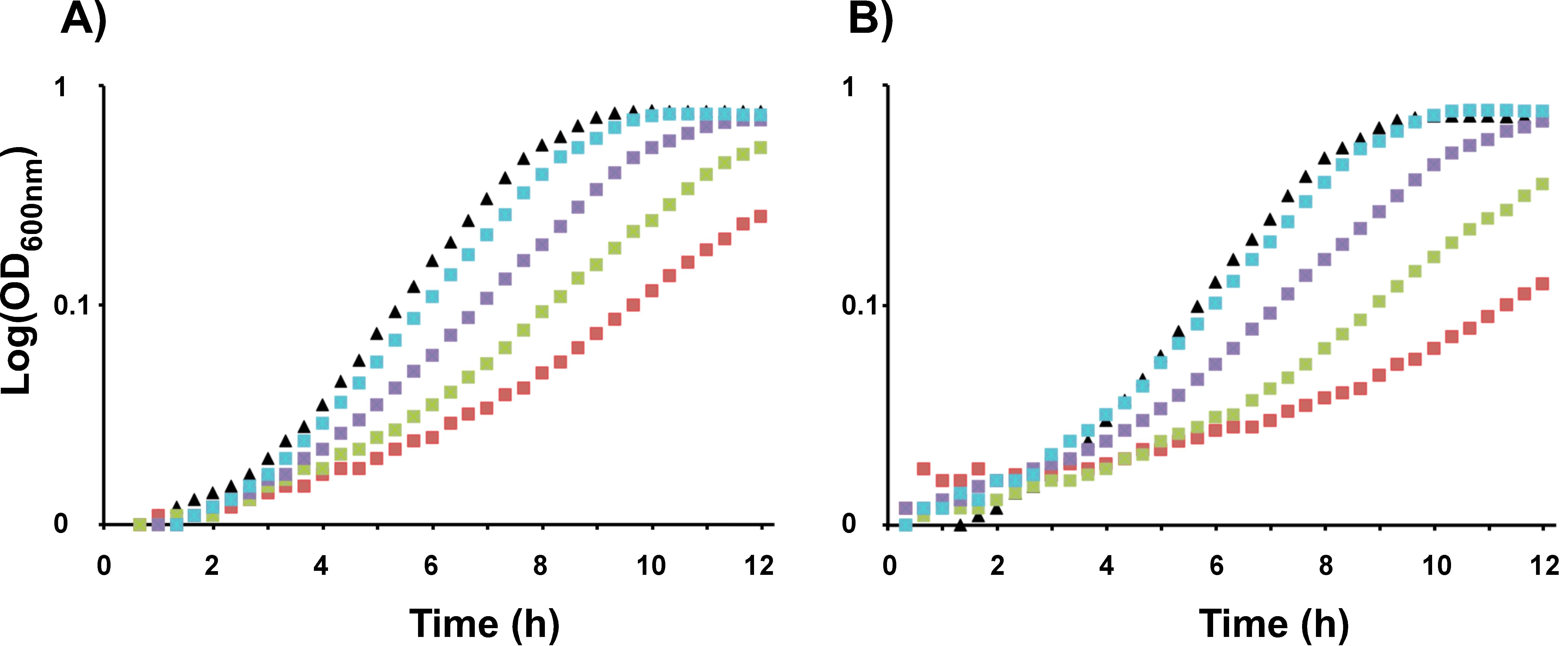


**Fig. S1.** Effect of the murine cathelicidin mCRAMP on bacterial growth. Overnight culture of the control strain *L. lactis* NZ9000/vec (A) or the *L. lactis* NZ9000/vecΩ*pilB* strain expressing the GBS PilB protein (B) were diluted in M17 broth supplemented with glucose (1%) and erythromycin (5 µg/ml) to give approximately 10^6^ cfu/ml. The inoculated broths were distributed (150 µl) in 96 wells plate incubated at 30°C with constant shaking in a plate reader and the OD_600nm_ was recorded every 20 minutes for 12 hours. Blank values (M17-glucose-erythromycin) were subtracted from experimental values to eliminate background readings. ▲, M17-glucose-erythromycin medium without peptide (sterile water was added instead); ■, presence of the mCRAMP drug at 5 (light blue), 10 (violet), 20 (green), and 40 (red) µg/ml , respectively. These results are representative of three independent experiments.
